# Inflammatory cytokines and distant recurrence in HER2-negative early breast cancer

**DOI:** 10.1038/s41523-021-00376-9

**Published:** 2022-02-08

**Authors:** Joseph A. Sparano, Anne O’Neill, Noah Graham, Donald W. Northfelt, Chau T. Dang, Antonio C. Wolff, George W. Sledge, Kathy D. Miller

**Affiliations:** 1 Icahn School of Medicine at Mount Sinai, Tisch Cancer Institute, New York, NY USA; 2 Dana Farber Cancer Institute ECOG-ACRIN Biostatistics Center, Boston, MA USA; 3 Mayo Clinic, Scottsdale, AZ USA; 4 Memorial Sloan Kettering Cancer Center, New York, NY USA; 5 Johns Hopkins University Sidney Kimmel Comprehensive Cancer Center, Baltimore, MD USA; 6 Stanford Cancer Center, Palo Alto, CA USA; 7 Indiana University Melvin and Bren Simon Comprehensive Cancer Center, Indianapolis, IN USA

**Keywords:** Breast cancer, Prognostic markers

## Abstract

Systemic inflammation is believed to contribute to the distant recurrence of breast cancer. We evaluated serum samples obtained at diagnosis from 249 case:control pairs with stage II-III Her2-negative breast cancer with or without subsequent distant recurrence. Conditional logistic regression analysis, with models fit via maximum likelihood, were used to estimate hazard ratios (HRs) and test for associations of cytokines with distant recurrence risk. The only biomarker associated with a significantly increased distant recurrence risk when adjusted for multiple testing was the proinflammatory cytokine IL-6 (HR 1.37, 95% confidence intervals [CI] 1.15, 1.65, *p* = 0.0006). This prospective-retrospective study provides evidence indicating that higher levels of the cytokine IL-6 at diagnosis are associated with a significantly higher distant recurrence risk.

## Introduction

Inflammation is a normal physiologic response to injury, including cancer^1^, and plays a key role in contributing to cancer progression^2,3^. Inflammation is mediated by cytokines, which are glycoproteins secreted by inflammatory cells that induce several intracellular kinases; chemokines are a subset cytokines that induce chemotaxis of other cells^4^. Although cytokines mediate inflammation, and inflammation facilitates cancer progression, no studies have evaluated the association between specific cytokines and recurrence of breast cancer.

Prior studies have indicated that elevated C-reactive protein (CRP) and serum amyloid A (SAA) are associated with an increased risk of breast cancer recurrence^5^. CRP and SAA are nonspecific, acute-phase hepatic proteins secreted in response to cytokines, providing indirect evidence that cytokines may be contributing to breast cancer recurrence. In order to determine which specific cytokines might be mediating this effect, we evaluated the association between distant recurrence of breast cancer and serum levels of 36 human cytokines and chemokines involved in chemotaxis, angiogenesis, and immune system regulation, including effector CD4 + Th1/Th2 and Th17 T cells. We evaluated the association between cytokine levels and distant recurrence in 249 case:control pairs (498 patients) after primary surgery and before systemic adjuvant chemotherapy, and in 17 case:control pairs (34 patients) about 5 years after diagnosis who were without clinical evidence of recurrence.

## Results

### Study population

**Fig. 1:**
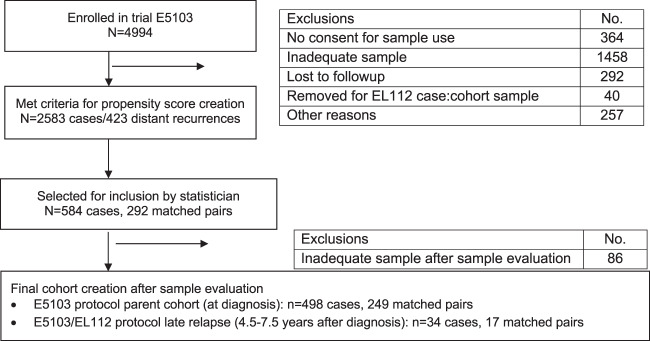
CONSORT diagram. Diagram indicating patients included and excluded from analysis and reasons for exclusion.

Of the 4994 patients enrolled on the parent E5103 trial, a total of 532 patients (10.7%) representing 266 case:control pairs were included in this analysis, including 498 patients representing 249 case:control pairs evaluating only a baseline specimen (which we refer to as parent cohort), plus 34 additional patients representing 17 case:control pairs without recurrence within the first 5 years enrolled in the late relapse EL112 substudy (which we refer to as the late relapse cohort) (Fig. 1, CONSORT Diagram). The distribution of covariates in the study populations are shown in Table 1. For the parent cohort, 88% were younger than 65 years, 46% were pre/peri-menopausal, 64% had hormone-receptor positive disease, 85% had at least one positive axillary node (including 53% with at least four positive axillary nodes), and 66% had tumors with poor histologic grade. Characteristics of the late relapse cohort were largely similar, except less likely to have ER- and PgR-negative disease (6% vs. 36%) and more likely to have low-grade tumors (18% vs. 3%).

**Table 1:** Baseline demographics and disease characteristics for factors used to create propensity score for matching at diagnosis and 5 years after diagnosis without recurrence within 5 years.

	Entire population	Parent cohort (at diagnosis)	Late relapse cohort (5 years after diagnosis)
Characteristics	Number (%) of patients	Number (%) of patients	Number (%) of patients
Total number			
Case:control pairs	Not applicable	249	17
Patients	4994	498	34
Age group (years)			
<40	627 (13)	74 (15)	–
40 to <65	3895 (78)	364 (73)	26 (76)
≥65	472 (9)	60 (12)	8 (24)
Menopausal status			
Pre/peri	2387 (48)	228 (46)	9 (26)
Post	2564 (52)	270 (54)	25 (74)
ERPgR status			
ER and PgR negative	1796 (36)	180 (36)	2 (6)
ER and/or PgR positive	3198 (64)	318 (64)	32 (94)
Tumor size			
≤2 cm	1933 (39)	83 (17)	6 (17)
>2 to ≤5 cm	2552 (51)	334 (67)	23 (68)
>5 cm	503 (10)	81 (16)	5 (15)
Nodal status			
Negative	1300 (26)	74 (15)	4 (12)
1–3	2139 (43)	157 (32)	5 (15)
4+	1555 (31)	267 (53)	25 (74)
Histologic grade			
I	422 (9)	15 (3)	6 (18)
II	1628 (33)	153 (31)	16 (47)
III	2833 (58)	330 (66)	12 (35)
Treatment^a^			
No bevacizumab	1000 (20)	96 (19)	5 (15)
Bevacizumab	3994 (80)	402 (81)	29 (85)

^a^Bevacizumab treatment was not a covariate used in propensity score matching; randomization to three arms in E5103 parent trial was 1:2:2 (placebo: bevacizumab × 4 doses; bevacizumab × 14 doses). The characteristics of both the parent cohort and late relapse cohort are those at diagnosis. Data were missing for ER/PR (*n* = 2) tumor size (*n* = 6), grade (*n* = 111), and menopausal status (*n* = 43, including 20 with male gender; all in case:control sample were female).

### Cytokine and chemokine levels at diagnosis and association with distant recurrence

Results for the cytokine and chemokine levels for the 498 patients in the parent cohort are shown in Supplementary Table 1, and for 34 patients in the late relapse cohort after 5 years in Supplementary Table 2. There were at least 200 informative case:control pairs (of a maximum of 249) for 23 of 36 cytokines for the parent cohort (Supplementary Table 3), and at least 11 informative case:control pairs (of a maximum of 17) for 25 of 36 cytokines for the late relapse cohort (Supplementary Table 4).

The only biomarker associated with a significantly increased distant recurrence risk when adjusted for multiple testing was the proinflammatory cytokine IL-6 (HR 1.37, 95% confidence intervals [CI] 1.15, 1.65, *p* = 0.0006; 232 case:control pairs). Others associated with a two-sided p value <0.05 (but exceeding 0.0014) included the T helper cell inflammatory cytokine IL-17A (HR 1.36, 95% CI 1.10, 1.67, *p* = 0.0052; 210 case:control pairs), included the chemokine MDC (macrophage-derived chemokine/CCL22) (HR 1.58, 95% CI 1.07, 2.33, *p* = 0.02; 249 case:control pairs), and the cytokine VEGF-A (HR 1.13, 95% CI 1.01, 1.27, *p* = 0.04; 240 case:control pairs) (Supplementary Table 3). There was no statistically significant interaction between elevated VEGF-A levels at diagnosis and benefit from adjuvant bevacizumab (*p*-value for interaction term = 0.34).

### Absolute cytokine/chemokine levels at diagnosis in parent cohort

The median and mean value (and range) in all patients in the parent cohort for the four biomarkers associated with distant recurrence was 0.95 and 7.53 pg/ml (0.04–2761.24 pg/ml) for IL-6, 3.32 and 4.04 pg/ml (0.12–43.11 pg/ml) for IL-17A, 1019.14 and 1081.84 pg/ml (392.86–4063.81 pg/ml) for MDC/CCL22, and 77.88 and 109.38 (0.66–1196.67 pg/mL) for VEGF-A (Supplementary Table 1). There was no significant difference in IL-6 level by histologic grade (Wilcoxon rank sum test comparing grade 1 versus grade 3, *p* = 0.62).

### Cytokines/chemokine levels at diagnosis and time to distant recurrence

**Fig. 2:**
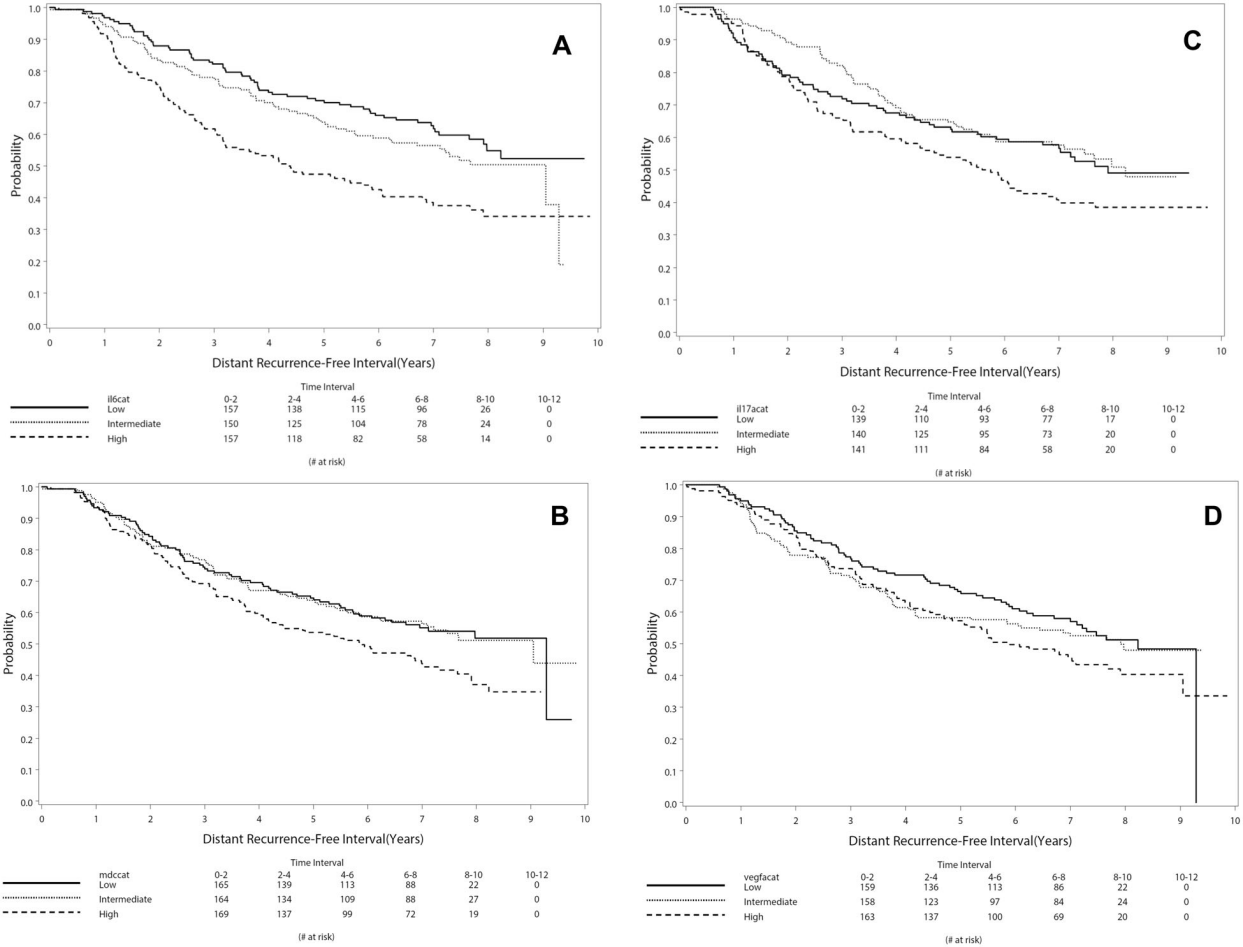
Time to event by tertile of IL-6, IL-17A, MDC/CCL22, and VEGF-A level. Kaplan–Meier plots of time to distant recurrence for (**A**) IL-6 (**B**), IL-17A, (**C**) MDC/CCL22, and (**D**) VEGF-A. All Kaplan–Meier plots show time to distant recurrence by low (≤33rd percentile), intermediate (34th–66th percentile), and high (>66th percentile) biomarker results.

Kaplan–Meier plots for time to distant recurrence are shown in Fig. 2A–D, including IL-6 (Fig. 2A), IL-17A (Fig. 2B), MDC/CCL22 (Fig. 2C), and VEGF-A (Fig. 2D), each showing an association with distant recurrence when evaluated by tertiles.

**Fig. 3:**
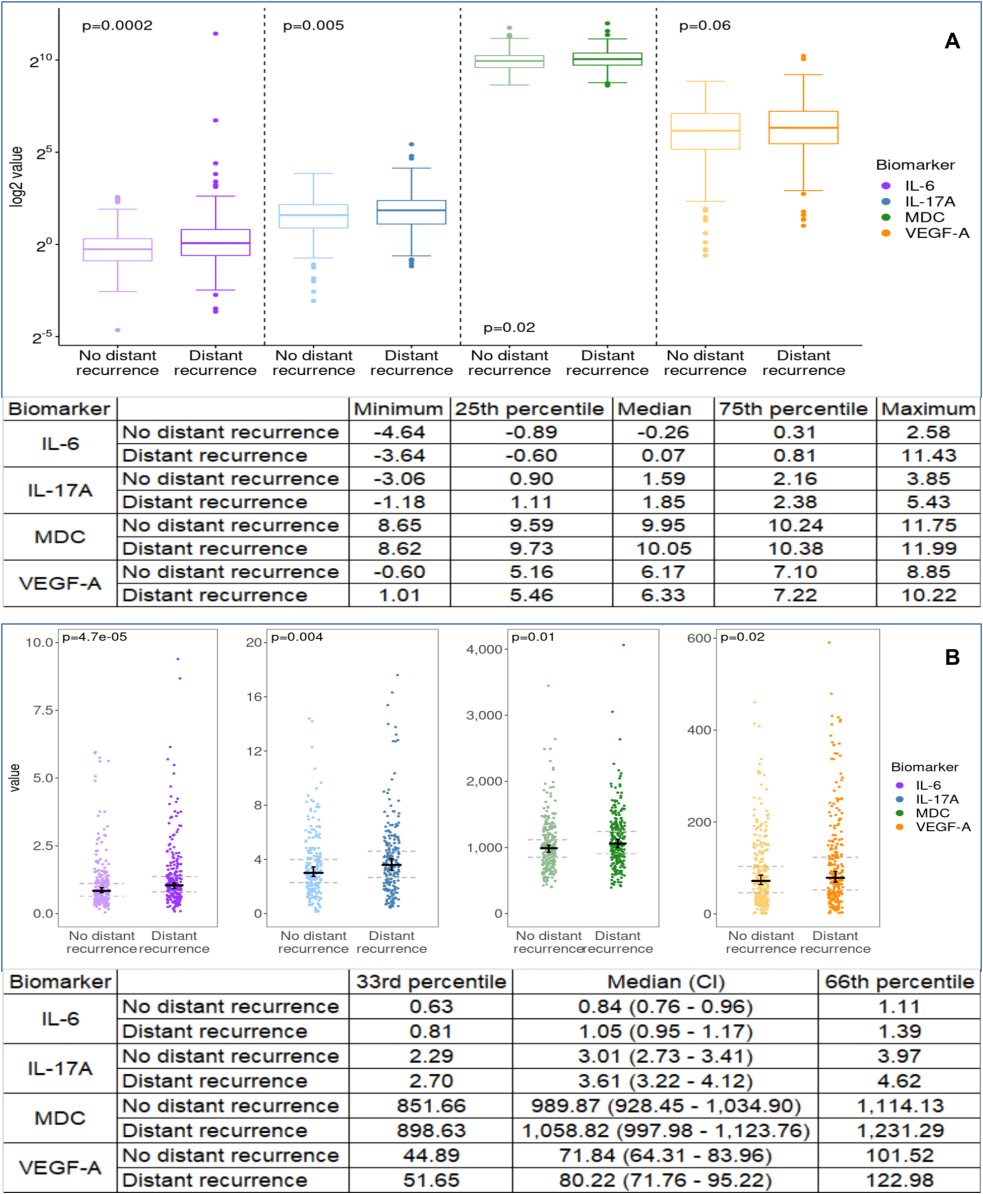
Blood levels of IL-6, IL-17A, MDC/CCL22, and VEGF-A. Box plots and actual values of log2value (**A**) for IL-6, IL-17A, MDC/CCL22, and VEGF-A in cases with distant recurrence compared with controls without distant recurrence. Dot plots and actual values of raw values (**B**) for IL-6, IL-17A, MDC/CCL22, and VEGF-A in cases with distant recurrence compared with controls without distant recurrence.

Box plots showing comparison of log2values in cases versus controls using Wilcoxon signed rank test revealed *p* values of 0.0002 for IL-6, 0.005 for IL-17A, 0.02 for MDC/CCL22, and 0.06 for VEGF-A (Fig. 3).

Dot plots showing comparison of raw values in cases versus controls using Wilcoxon signed rank test revealed p values of 4.7e-05 for IL-6, 0.004 for IL-17A, 0.01 for MDC/CCL22, and 0.02 for VEGF-A.

### Association between body mass index at diagnosis and cytokines/chemokines

Of the 498 patients in the parent cohort, 41% (205/498) were obese (BMI ≥ 30 kg/m^2^), 33% were overweight (BMI 25–29.9 kg/m^2^), and 26% had a normal BMI at trial entry. High baseline BMI (≥30 kg/m^2^) was associated with significantly higher IL-6 (*p* < 0.01) and MDC/CCL22 levels (*p* = 0.03), but not with IL-17A (*p* = 0.18) or VEGF-A (*p* = 0.64) (Supplementary Table 5). There was no significant difference in BMI at trial entry between controls and cases (mean 29.2 versus and 30.4 kg/m^2,^ *p* = 0.12).

### Cytokine and chemokine levels at 5 years and association with distant recurrence

At ~5 years after diagnosis in 34 patients (17 case:control pairs) in the late relapse cohort, exploratory analysis did not identify any biomarker significantly associated with recurrence (Supplementary Table 4), including IL-6 (HR 1.81, 95% CI 0.75, 4.37, *p* = 0.19, 17 case-control pairs), IL-17A (HR 1.06, 95% CI 0.61, 1.84, *p* = 0.83, 14 case:control pairs), MDC/CCL22 (HR 1.12, 95% CI 0.19, 6.53, *p* = 0.89, 17 case:control pairs), and VEGF-A (HR 0.78, 95% CI 0.39, 1,53, *p* = 0.46, 17 case:control pairs). There was no significant difference in IL-6 level by histologic grade (Wilcoxon rank sum test comparing grade 1 versus grade 3, *p* = 0.60).

## Discussion

We performed a case:control study including a total of 532 patients (including 266 case:control pairs) with high-risk stage II–III HER2-negative breast cancer to determine whether one or more serum cytokine levels at diagnosis, or 5 years after diagnosis when cancer-free, was associated with distant breast cancer recurrence despite surgery and adjuvant chemotherapy (plus endocrine therapy if ER-positive disease). We employed a discovery-based panel of 36 human cytokines and chemokines selected based on evidence for their role in chemotaxis, angiogenesis, and immune system regulation. Four cytokines measured at diagnosis were associated with distant recurrence, including the cytokine IL-6, IL-17A, and VEGF-A, and the chemokine MDC/CCL22. This prospective:retrospective study performed in accordance with REMARK biomarker guidelines^6^ demonstrates an association between serum cytokine levels and distant breast cancer recurrence, providing a foundation for additional validation of these findings in other cohorts. Similar associations were not observed when blood samples were evaluated 5 years after diagnosis in those who were recurrence-free and had later recurrence, although the sample size was limited, and less than planned.

Of the four cytokines associated with distant recurrence, IL-6 had the most robust association, and the only one whose association was statistically significant after adjustment for multiple testing (HR 1.37, *p* = 0.0006). The IL-6/JAK/STAT3 pathway has a key role in the growth and development of many human cancers. Elevated levels of IL-6 are observed in chronic inflammatory conditions, such as rheumatoid arthritis and inflammatory bowel disease, and in a variety of cancers^7^, including breast cancer^8^. IL-6 stimulates hyperactivation of JAK/STAT3 signaling, which promotes tumor proliferation, angiogenesis, invasiveness, and immunosuppression^9^. There are currently two approved agents targeting IL-6, including antibodies targeting IL-6 (siltuximab) or the IL-6 receptor (tocilizumab). Siltuximab is chimeric mouse–human antibody approved for the treatment of multicentric Castleman disease^10^. Tocilizumab is a humanized monoclonal antibody approved for the treatment of systemic juvenile idiopathic arthritis and cytokine-release syndrome occurring in adults or children after CAR-T cells^11,12^. A number of trials are ongoing evaluating the combination of IL-6 antibodies plus cytotoxic therapy in a variety of cancer types^9^. Evaluation of such combinations in the neoadjuvant setting, with pathologic response as the endpoint as in the I-SPY trial^13^, may be a reasonable strategy to determine whether high IL-6 levels identify a population that may benefit targeting the IL-6 axis, and whether larger scale trials are warranted.

Three other cytokines demonstrated an association with distant recurrence, including IL-17A, macrophage-derived chemokine/CCL22, VEGF-A, though not statistically significant after adjustment for multiple testing. IL-17A is a pro-inflammatory cytokine that plays critical role in host defenses on barrier surfaces, and plays a role in autoimmune conditions^14^. The primary function of Th17 cells that secrete IL-17A includes control of the gut microbiota and clearance of extracellular bacteria and fungi^14^. Several monoclonal antibodies that inhibit the function of IL-17A have been approved for psoriasis and psoriatic arthritis (secukinumab, ixekizumab, brodulmab)^15^. Evidence indicates that IL-17A may also have a role in promoting metastasis by eliciting expansion and polarization of neutrophils that suppress cytotoxic CD8 T lymphocytes in mouse mammary tumors, thereby promoting metastasis, which may be abrogated by neutralization of IL-17A^16^. IL-17A can also enhance tumor growth in vivo through the induction of IL-6^17^. Chemokines such as MDC/CCL22 provide directional cues during migration of leukocytes to damaged or infected tissues, including cancer^18^. Finally, VEGF-A is a cytokine that facilitates growth of new blood vessels. The anti-VEGF antibody has been extensively evaluated for the treatment of metastatic breast cancer, where it modestly prolongs progression-free survival when added to chemotherapy, but did not improve overall survival^19^. In metastatic breast cancer, pretreatment plasma VEGF-A level was not predictive of benefit from bevacizumab^20^. In addition, adjuvant bevacizumab was not effective in reducing distant recurrence risk when added to adjuvant chemotherapy^21^. This experience with VEFG-A as a biomarker for predicting benefit from bevacizumab highlights the limitations of this approach with the other potentially promising biomarkers identified in our study.

Obesity has been associated with increased recurrence risk in localized estrogen-receptor positive breast cancer^22,23^. Although higher BMI was associated with significantly higher IL-6 (*p* < 0.01) and MDC/CCL22 levels (*p* = 0.03), there was no significant difference in mean BMI in cases with recurrence compared with controls without recurrence. Further evaluation of IL-6 and MDC/CCL22 as prognostic biomarkers should include evaluation of BMI as a confounding factor.

In conclusion, higher serum levels of several cytokines at diagnosis was associated with an increased distant recurrence risk in localized breast cancer, providing a foundation for further assessment and prospective validation of IL-6, IL-17A, and MDC/CCL22 as prognostic biomarkers, and IL-6 as a candidate predictive marker for therapeutic strategies targeting the IL-6/JAK/STAT3 pathway. Strengths of the analysis include the prospective-retrospective study design conducted in accordance with REMARK guidelines and use of a multiplex cytokine panel including selected biomarkers, whereas limitations include lack of validation in an independent cohort, and inadequate sample size for the late relapse cohort.

## Methods

### Eligibility criteria

The study population included patients enrolled in a previously completed E5103 adjuvant clinical trial (NCT00433511), which accrued 4994 patients with node-positive or high-risk node-negative, human epidermal growth factor receptor 2 (Her2)-negative operable breast cancer between November 2007 and February 2011. The protocol stipulated adjuvant therapy including sequential doxorubicin/cyclophosphamide-paclitaxel chemotherapy alone or in combination with bevacizumab, plus endocrine therapy for patients with hormone receptor-positive disease. Five-year invasive disease-free survival rates ranged from 76–80%, and were not significantly impacted by bevacizumab use^21^. Trial participants were eligible to participate in a late recurrence substudy (EL112) if they were without clinical evidence of recurrence based on history and physical exam between 4.5 and 7.5 years after registration in the original E5103 trial and consented to participate in the substudy.

### Study design and statistical analysis plan

This prospective-retrospective study was designed and results reported in accordance with the REMARK guidelines^6^. The primary endpoint was time to distant recurrence, defined as date from registration to first invasive distant recurrence; patients without documentation of invasive distant recurrence were censored at date last known disease-free. Clinical recurrences were determined by the local treating physician based on clinical, radiographic, and pathologic information, and confirmed by a research coordinator in the ECOG-ACRIN data management center and study principal investigator (KDM). Propensity score matching was used to identify ~250 case: control pairs with and without distant recurrence*.* The covariates used to create propensity score matching included age (<40(referent), 40–64, ≥65 years), menopausal status (pre/peri vs. post), estrogen/progesterone receptor expression (both negative vs. one or both positive), tumor size (≤2 cm(referent), 2–5 cm, >5 cm), axillary nodal status (negative(referent), 1–3 positive, ≥4 positive), and histologic grade (I (referent), II, III).

Conditional logistic regression analysis, with models fit via maximum likelihood, were used to estimate hazard ratios (HR) and test for associations. With ~250 matched pairs, it was estimated that there was 80% power to detect a HR of 1.66 using methods previously described^24^. As an additional exploratory aim, we evaluated an additional 17 case-control pairs (of 24 planned to provide 80% power to detect a HR of 6.45) including patients without recurrence within the first 4.5–7.5 years after diagnosis who provided a blood specimen a part of the EL112 substudy. Due to skewed nature of cytokines, HRs are reported on Log2 (log base 2); therefore, a two-fold elevation in the original value of a marker was associated with the stated HR for distant recurrence. The Kaplan–Meier method was used to estimate time to recurrence distributions by tertiles. Wilcoxon test as appropriate or Kruskal Wallis test (>2 category variable) was used to test for associations between markers and baseline demographic and disease characteristic categorical variables. Note that the results for markers are reported among the informative pairs (where marker values were available for the case and the control within the matched pair). Given multiple comparisons across many cytokines with relatively few informative case:control pairs, HR estimates with *p*-value < 0.002 would be considered for statistical significance (Bonferroni adjustment: 0.05/23 for 23 of 36 cytokines with ≥200 informative pairs). If considering all 36 markers, a *p*-value of <0.0014 would be required for statistical significance. Because of the association between obesity and inflammation, the association between body mass index with cytokine values was also evaluated. All statistical analyses were performed using SAS9.4 and R 3.6.1. For the graph in Fig. 3B, a total of 10 pairs (20 patients) were excluded due to outlying values outside of the ranges used in the graph, including 5 pairs for IL6, 3 pairs for IL17A, and pairs from VEGF-A; these values are included in the table appearing in Fig. 3B, and also included in the statistical comparison.

### Cytokine/chemokine assay

Serum samples were collected at the time of trial registration after potentially curative primary surgery, and before systemic adjuvant therapy. Archived frozen serum samples stored in the ECOG-ACRIN Central Biorepository and Pathology Facility (CBPF) at the MD Anderson Cancer Center were analyzed using the MSD V-Plex Human Cytokine 36-Plex Kit (https://www.mesoscale.com/products/v-plex-plus-human-cytokine-36-plex-kit-k15089g) for detection of a panel of 36 human cytokines and chemokines, including the following: Eotaxin, Eotaxin-3, GM-CSF, IFN-γ, IL-10, IL-12/IL-23p40, IL-12p70, IL-13, IL-15, IL-16, IL-17A, IL-1α, IL-1β, IL-2, IL-21, IL-22, IL-23, IL-27, IL-31, IL-4, IL-5, IL-6, IL-7, IL-8, IL-8 (HA), IP-10, MCP-1, MCP-4, MDC, MIP-1α, MIP-1β, MIP-3α, TARC, TNF-α, TNF-β, VEGF-A. Samples were analyzed in accordance with the manufacturer’s instructions by the Translational Molecular Pathology and Immunoprofiling Lab Oncology Research and Immuno-monitoring Core at MD Anderson Cancer Center.

### Study oversight

The first author (J.A.S.) wrote the manuscript, the second and third authors (A.O., N.G.) performed the statistical analysis, and the final version incorporated changes recommended by the coauthors. Data were collected by the ECOG–ACRIN Cancer Research Group Coordinating Center. All the authors vouch for the accuracy and completeness of the data and analyses presented and for the adherence of the study to the protocol. No commercial support was involved in the planning or execution of this secondary analysis of the E5103 trial. The parent E5103 study and EL112 late relapse substudy were reviewed and approved by the Cancer Therapy Evaluation Program, National Cancer Institute, and by the local institutional review board at each participating center.

### Reporting summary

Further information on research design is available in the Nature Research Reporting Summary linked to this article.

## Supplementary information

Supplementary Information

Reporting Summary

## Data Availability

The datasets generated during and/or analyzed during the current study are available in the NCTN/NCORP Data Archive (https://nctn-data-archive.nci.nih.gov).
