# Supplementary material for: Inflammatory cytokines and distant recurrence in HER2-negative early breast cancer
Source: NPJ Breast Cancer. 2022 Feb 8;8:16. doi: 10.1038/s41523-021-00376-9 (PMC8825796; doi:10.1038/s41523-021-00376-9)
Supplement: Supplementary file 1 — Supplementary Information [file 41523_2021_376_MOESM1_ESM.pdf]

**Supplementary Information**

Supplement to: Inflammatory cytokines and distant recurrence in HER2-negative early breast cancer

**Supplementary Table 1.** Results of inflammatory cytokine and chemokine analysis at diagnosis (parent cohort) (n=498 patients)

**Supplementary Table 2.** Results of inflammatory cytokine and chemokine analysis at five years after diagnosis (late relapse cohort) (n=34 patients)

**Supplementary Table 3.** Results of conditional logistic regression analysis, with models fit via maximum likelihood, to estimate hazard ratios and test for associations between distant recurrence and cytokine/chemokine levels at diagnosis (parent cohort) (n=498 patients)

**Supplementary Table 4.** Results of conditional logistic regression analysis, with models fit via maximum likelihood, to estimate hazard ratios and test for associations between distant recurrence and cytokine/chemokine levels five years after diagnosis (late relapse cohort) (n=34 patients)

**Supplementary Table 5.** Association between body mass index and cytokines (parent cohort)

**Supplementary Table 1.** Descriptive statistics of inflammatory cytokine and chemokine analysis at diagnosis (parent cohort) (n=498 patients) (among matched pairs with cytokine values for both the case and the control)

| | N | Mode | Median | Mean | Minimum | Maximum | Std Dev | Skewness |
|---|---|---|---|---|---|---|---|---|
| Eotaxin | 498 | 108.7600 | 171.45000 | 195.51226 | 13.69000 | 860.48000 | 110.80523 | 1.535748 |
| Eotaxin3 | 400 | 2.740000 | 7.0550000 | 89.207800 | 0.430000 | 28134.99 | 1408.86 | 19.87331 |
| GMCSF | 94 | 0.060000 | 0.1400000 | 0.1765957 | 0.020000 | 0.9500000 | 0.165058 | 2.675150 |
| IFNy | 424 | 2.080000 | 3.1200000 | 5.0003538 | 0.250000 | 84.280000 | 7.709447 | 6.456080 |
| IL10 | 482 | 0.180000 | 0.270000 | 0.3719295 | 0.020000 | 6.4900000 | 0.4597064 | 7.152379 |
| IL12IL23p40 | 498 | 77.4600 | 110.870 | 128.2738 | 2.430000 | 594.62000 | 72.44559 | 1.482034 |
| Il12p70 | 176 | 0.090000 | 0.135000 | 0.4081250 | 0.020000 | 15.07000 | 1.3971577 | 8.184657 |
| lL13 | 128 | 0.280000 | 0.590000 | 0.8599219 | 0.030000 | 24.990000 | 2.1937228 | 10.64551 |
| IL15 | 498 | 2.33000 | 2.53500 | 2.584618 | 1.410000 | 5.2100000 | 0.587737 | 0.772443 |
| IL16 | 498 | 267.830 | 258.750 | 320.9342 | 56.57000 | 14117.46 | 662.2464 | 18.64273 |
| IL17A | 420 | 0.630000 | 3.315000 | 4.0421429 | 0.120000 | 43.110000 | 3.6670428 | 4.669457 |
| IL17AGenB | 44 | 0.420000 | 1.155000 | 1.9313636 | 0.040000 | 19.900000 | 3.0642512 | 4.949988 |
| IL1α | 31 | 0.06000 | 0.19000 | 0.682580 | 0.010000 | 10.920000 | 1.951542 | 5.148765 |
| IL1β | 346 | 0.050000 | 0.080000 | 5.5771098 | 0.010000 | 1868.61 | 100.45063 | 18.59937 |
| IL2 | 168 | 0.070000 | 0.135000 | 0.3181071 | 0.001000 | 22.170000 | 1.7062094 | 12.73055 |
| IL21 | 16 | 0.220000 | 0.440000 | 2.4425000 | 0.040000 | 20.13000 | 4.9759602 | 3.37505 |
| IL22 | 310 | 0.710000 | 0.870000 | 1.6135161 | 0.110000 | 27.930000 | 3.0616275 | 5.680694 |
| IL23 | 8 | . | 2.095000 | 5.4200000 | 0.970000 | 28.790000 | 9.5029394 | 2.758194 |
| IL27 | 498 | 1119.55 | 1322.38 | 1533.01 | 195.3500 | 74871.75 | 3350.89 | 21.19555 |
| IL31 | 116 | 0.070000 | 0.125000 | 0.2224138 | 0.010000 | 4.1300000 | 0.4532887 | 6.857028 |
| IL4 | 204 | 0.010000 | 0.030000 | 0.1400490 | 0.001000 | 19.890000 | 1.3904647 | 14.25 |
| IL5 | 130 | 0.140000 | 0.230000 | 0.3819308 | 0.001000 | 2.1900000 | 0.4089982 | 2.317127 |
| IL6 | 464 | 0.560000 | 0.950000 | 7.5343534 | 0.040000 | 2761.24 | 128.21613 | 21.49002 |
| IL7 | 498 | 13.0700 | 13.9850 | 14.71042 | 1.270000 | 48.870000 | 7.148840 | 0.892999 |
| IL8 | 498 | 11.2400 | 11.5050 | 20.48202 | 2.150000 | 2348.40 | 110.1147 | 19.52253 |
| IP10 | 498 | 191.050 | 251.525 | 301.1576 | 1.460000 | 4485.91 | 260.1648 | 9.172807 |
| MCP1 | 498 | 138.440 | 246.160 | 266.6054 | 34.70000 | 1711.32 | 131.1619 | 3.339569 |
| MCP4 | 498 | 74.6900 | 95.6950 | 115.0175 | 3.460000 | 878.68000 | 88.98606 | 3.470259 |
| MDC | 498 | 1058.58 | 1019.14 | 1081.04 | 392.8600 | 4063.81 | 431.4663 | 1.825807 |
| MIP1α | 458 | 9.870000 | 11.43500 | 21.475982 | 1.160000 | 2048.22 | 113.03554 | 15.55300 |
| MIP1β | 498 | 67.10000 | 96.42000 | 110.03979 | 0.580000 | 1564.15 | 85.515600 | 10.37171 |
| MIp3α | 482 | 2.540000 | 5.540000 | 8.2526763 | 0.310000 | 253.0800 | 15.507001 | 11.22741 |
| TARC | 496 | 68.13000 | 190.0300 | 245.24895 | 1.970000 | 1779.09 | 213.03745 | 2.531856 |
| TNFα | 498 | 1.92000 | 2.04000 | 2.462590 | 0.230000 | 85.040000 | 4.475397 | 14.92036 |
| TNFβ | 474 | 0.440000 | 0.395000 | 0.4363502 | 0.030000 | 4.4600000 | 0.2820830 | 6.784017 |
| VEGFA | 480 | 20.79000 | 77.87500 | 109.37602 | 0.660000 | 1196.67 | 114.76849 | 3.839740 |

**Supplementary Table 2.** Descriptive Statistics of inflammatory cytokine and chemokine analysis at five years after diagnosis (late relapse cohort) (n=34 patients) (among matched pairs with cytokine values for both the case and the control)

| | N | Mode | Median | Mean | Minimum | Maximum | Std Dev | Skewness |
|---|---|---|---|---|---|---|---|---|
| Eotaxin | 34 | . | 208.92500 | 246.02705 | 107.87000 | 1096.22 | 171.39281 | 3.92533 |
| Eotaxin3 | 32 | 8.04000 | 8.2400000 | 19.574062 | 1.9900000 | 145.30000 | 28.389345 | 3.21555 |
| GMCSF | 4 | . | 0.8350000 | 1.0150000 | 0.2000000 | 2.1900000 | 0.9732591 | 0.48733 |
| IFNy | 30 | 1.06000 | 4.8150000 | 24.152333 | 1.0600000 | 272.50000 | 66.343784 | 3.60527 |
| IL10 | 28 | 0.34000 | 0.5950000 | 0.7250000 | 0.0500000 | 3.8000000 | 0.7315610 | 3.11744 |
| IL12IL23p40 | 34 | . | 128.37000 | 168.63529 | 59.050000 | 535.18000 | 103.93906 | 1.85583 |
| IL12p70 | 26 | 0.09000 | 0.3500000 | 0.8773077 | 0.0800000 | 8.7900000 | 1.7248642 | 4.21125 |
| IL13 | 22 | . | 1.7950000 | 2.6045455 | 0.3100000 | 13.430000 | 2.8909625 | 2.78918 |
| IL15 | 34 | 3.97000 | 4.3100000 | 4.6976471 | 3.3000000 | 9.9700000 | 1.3508360 | 2.32209 |
| IL16 | 34 | . | 339.92000 | 351.86941 | 163.53000 | 568.36000 | 106.53979 | 0.39342 |
| IL17A | 28 | . | 5.2900000 | 7.1307143 | 1.1000000 | 22.960000 | 5.5211680 | 1.53264 |
| IL17AGenB | 0 | | | | | | | |
| IL1α | 2 | . | 1.2150000 | 1.2150000 | 0.0800000 | 2.3500000 | 1.6051324 | . |
| IL1β | 4 | . | 0.1650000 | 0.4575000 | 0.0600000 | 1.4400000 | 0.6569817 | 1.96344 |
| Il2 | 12 | . | 0.4350000 | 0.6450000 | 0.0600000 | 2.7100000 | 0.7441957 | 2.33240 |
| IL21 | 2 | . | 2.8650000 | 2.8650000 | 1.3700000 | 4.3600000 | 2.1142493 | . |
| IL22 | 8 | . | 1.8350000 | 3.2600000 | 0.3600000 | 10.860000 | 3.7108913 | 1.62900 |
| IL23 | 0 | | | | | | | |
| IL27 | 34 | . | 1315.69 | 1381.90 | 426.92000 | 3144.67 | 600.60614 | 0.84922 |
| IL31 | 0 | | | | | | | |
| IL4 | 18 | 0.04000 | 0.0500000 | 0.0589444 | 0.0010000 | 0.1800000 | 0.0404831 | 1.58079 |
| IL5 | 12 | . | 0.7100000 | 1.3041667 | 0.0800000 | 6.9600000 | 1.8902403 | 2.82743 |
| IL6 | 34 | 1.07000 | 1.5800000 | 2.0252941 | 0.4300000 | 6.9400000 | 1.4434979 | 1.57424 |
| IL7 | 34 | . | 20.575000 | 24.03500 | 3.0700000 | 66.410000 | 13.52288 | 1.25012 |
| IL8 | 34 | . | 12.925000 | 16.871764 | 4.0800000 | 79.820000 | 13.553877 | 3.32236 |
| IP10 | 34 | . | 307.26000 | 337.48294 | 60.940000 | 938.75000 | 225.78708 | 1.26472 |
| MCP1 | 34 | . | 338.07000 | 351.60352 | 134.19000 | 585.80000 | 118.93339 | 0.32795 |
| MCP4 | 34 | . | 149.96000 | 205.19588 | 54.710000 | 768.00000 | 167.2356 | 2.03469 |
| MDC | 34 | . | 1332.39 | 1423.43 | 889.81000 | 2879.89 | 434.31411 | 1.32121 |
| MIP1α | 32 | 16.0700 | 19.505000 | 20.778750 | 9.3500000 | 37.490000 | 5.7639501 | 0.80481 |
| MIP1β | 34 | . | 109.05000 | 114.82529 | 25.020000 | 230.64000 | 46.864557 | 0.53902 |
| MIP3α | 34 | . | 5.9650000 | 17.207352 | 1.8300000 | 239.13000 | 41.73742 | 4.89083 |
| TARC | 34 | . | 291.06500 | 376.08500 | 87.990000 | 1027.94 | 237.40724 | 1.15845 |
| TNFα | 32 | 4.80000 | 2.9850000 | 3.1550000 | 1.1900000 | 8.0000000 | 1.5655526 | 1.36868 |
| TNFβ | 34 | 0.46000 | 0.5600000 | 0.6785294 | 0.1200000 | 4.1500000 | 0.6396123 | 5.10841 |
| VEGFA | 34 | . | 131.63000 | 147.70029 | 21.830000 | 345.55000 | 86.343284 | 0.75977 |

**Supplementary Table 3.** Results of conditional logistic regression analysis, with models fit via maximum likelihood, to estimate hazard ratios and test for associations between distant recurrence and cytokine/chemokine levels at diagnosis (parent cohort) (n=498 patients)

| Biomarker | HR* | LL .95CI | UL .95CI | p-value | N | N observations (informative) | N pairs used (informative) |
|---|---|---|---|---|---|---|---|
| IL6 | 1.374 | 1.147 | 1.646 | 0.0006 | 477 | 464 | 232 |
| IL17a | 1.360 | 1.096 | 1.688 | 0.0052 | 448 | 420 | 210 |
| MDC | 1.578 | 1.068 | 2.331 | 0.02 | 498 | 498 | 249 |
| VEGFA** | 1.132 | 1.008 | 1.272 | 0.04 | 489 | 480 | 240 |
| Eotaxin | 0.825 | 0.656 | 1.038 | 0.10 | 498 | 498 | 249 |
| Eotaxin3 | 0.931 | 0.804 | 1.079 | 0.34 | 437 | 400 | 200 |
| IFNy | 1.161 | 0.979 | 1.377 | 0.09 | 455 | 424 | 212 |
| iL10 | 1.189 | 0.971 | 1.457 | 0.09 | 490 | 482 | 241 |
| IL12IL23p40 | 1.049 | 0.852 | 1.291 | 0.65 | 498 | 498 | 249 |
| IL15 | 0.834 | 0.451 | 1.542 | 0.53 | 498 | 498 | 249 |
| IL16 | 1.185 | 0.904 | 1.554 | 0.22 | 498 | 498 | 249 |
| IL27 | 1.155 | 0.846 | 1.576 | 0.36 | 498 | 498 | 249 |
| IL7 | 1.013 | 0.801 | 1.282 | 0.91 | 498 | 498 | 249 |
| IL8 | 1.148 | 0.941 | 1.401 | 0.17 | 498 | 498 | 249 |
| IP10 | 1.004 | 0.798 | 1.263 | 0.97 | 498 | 498 | 249 |
| MCP1 | 1.091 | 0.832 | 1.430 | 0.53 | 498 | 498 | 249 |
| MCP4 | 0.954 | 0.786 | 1.158 | 0.63 | 498 | 498 | 249 |
| MIP1α | 1.051 | 0.855 | 1.293 | 0.64 | 477 | 458 | 229 |
| MIP1β | 0.986 | 0.790 | 1.230 | 0.89 | 498 | 498 | 249 |
| MIP3α | 1.069 | 0.908 | 1.259 | 0.42 | 490 | 482 | 241 |
| TARC | 1.065 | 0.920 | 1.232 | 0.39 | 497 | 496 | 248 |
| TNFα | 1.256 | 0.933 | 1.692 | 0.13 | 498 | 498 | 249 |
| TNFβ | 1.159 | 0.892 | 1.504 | 0.27 | 479 | 474 | 237 |
| IL1β | 1.114 | 0.937 | 1.324 | 0.22 | 396 | 346 | 173 |
| IL22 | 1.167 | 0.970 | 1.405 | 0.10 | 391 | 310 | 155 |
| IL4 | 1.138 | 0.929 | 1.393 | 0.21 | 290 | 204 | 52 |
| GMCSF | 1.383 | 0.893 | 2.142 | 0.15 | 165 | 94 | 47 |
| IL12p70 | 0.816 | 0.637 | 1.046 | 0.11 | 240 | 176 | 88 |
| IL13 | 1.496 | 0.978 | 2.286 | 0.06 | 225 | 128 | 64 |
| Il17AGenB | 1.420 | 0.918 | 2.197 | 0.12 | 124 | 44 | 22 |
| IL1α | No estimate | | | | | | |
| IL2 | 1.111 | 0.900 | 1.371 | 0.33 | 265 | 168 | 84 |
| IL21 | 0.860 | 0.377 | 1.958 | 0.72 | 68 | 16 | 8 |
| IL23 | No estimate | | | | | | |
| IL31 | 0.825 | 0.612 | 1.111 | 0.20 | 229 | 116 | 58 |
| IL5 | 1.235 | 0.937 | 1.628 | 0.13 | 256 | 130 | 65 |

*Due to skewed nature of cytokines, HRs are reported on Log2 (log base 2) scale.
**there was no statistically significant interaction between treatment (bevacizumab vs no bevacizumab) and VEGFA (p-value for interaction term=0.34).

**Supplementary Table 4.** Results of conditional logistic regression analysis, with models fit via maximum likelihood, to estimate hazard ratios and test for associations between distant recurrence and cytokine/chemokine levels five years after diagnosis (late relapse cohort) (n=34 patients)

| Biomarker | HR* | LL .95CI | UL .95CI | p-value | N | N observations Used (informative) | N pairs used |
|---|---|---|---|---|---|---|---|
| IL6 | 1.808 | 0.748 | 4.372 | 0.19 | 34 | 34 | 17 |
| IL17a | 1.063 | 0.613 | 1.843 | 0.83 | 31 | 28 | 14 |
| MDC | 1.123 | 0.193 | 6.525 | 0.89 | 34 | 34 | 17 |
| VEGFA | 0.775 | 0.392 | 1.531 | 0.46 | 34 | 34 | 17 |
| Eotaxin | 1.264 | 0.469 | 3.408 | 0.64 | 34 | 34 | 17 |
| Eotaxin3 | 1.913 | 0.788 | 4.647 | 0.15 | 33 | 32 | 16 |
| IFNy | 1.115 | 0.779 | 1.596 | 0.55 | 32 | 30 | 15 |
| IL10 | 1.383 | 0.554 | 3.454 | 0.49 | 31 | 28 | 14 |
| IL12IL23p40 | 0.956 | 0.371 | 2.462 | 0.93 | 34 | 34 | 17 |
| IL15 | 1.113 | 0.146 | 8.45 | 0.92 | 34 | 34 | 17 |
| Il16 | 2.585 | 0.499 | 13.382 | 0.26 | 34 | 34 | 17 |
| Il27 | 0.350 | 0.105 | 1.173 | 0.09 | 34 | 34 | 17 |
| Il7 | 1.067 | 0.536 | 2.122 | 0.85 | 34 | 34 | 17 |
| IL8 | 1.239 | 0.573 | 2.679 | 0.59 | 34 | 34 | 17 |
| IP10 | 1.101 | 0.617 | 1.967 | 0.74 | 34 | 34 | 17 |
| MCP1 | 0.816 | 0.220 | 3.029 | 0.76 | 34 | 34 | 17 |
| MCP4 | 1.059 | 0.514 | 2.179 | 0.88 | 34 | 34 | 17 |
| MIP1α | 1.274 | 0.253 | 6.415 | 0.79 | 33 | 32 | 16 |
| MIP1β | 1.185 | 0.424 | 3.315 | 0.75 | 34 | 34 | 17 |
| MIP3α | 0.987 | 0.612 | 1.590 | 0.96 | 34 | 34 | 17 |
| TARC | 0.888 | 0.424 | 1.860 | 0.75 | 34 | 34 | 17 |
| TNFα | 1.580 | 0.536 | 4.658 | 0.41 | 33 | 32 | 16 |
| TNFβ | 1.229 | 0.422 | 3.580 | 0.70 | 34 | 34 | 17 |
| IL1β | 1.308 | 0.406 | 4.212 | 0.65 | 5 | 4 | 2 |
| IL22 | 1.154 | 0.395 | 3.376 | 0.79 | 14 | 8 | 4 |
| IL4 | 3.425 | 0.476 | 24.617 | 0.22 | 24 | 18 | 9 |
| GMCSF | No estimate | | | | 16 | 4 | 2 |
| IL12p70 | 1.765 | 0.752 | 4.140 | 0.19 | 30 | 26 | 13 |
| IL13 | 1.097 | 0.412 | 2.917 | 0.85 | 27 | 22 | 11 |
| IL17AGenB | No estimate | | | | 6 | 0 | 0 |
| IL1α | No estimate | | | | 9 | 2 | 1 |
| IL2 | 0.650 | 0.257 | 1.649 | 0.36 | 21 | 12 | 6 |
| IL21 | No estimate | | | | 8 | 2 | 1 |
| IL23 | No estimate | | | | 0 | | |
| IL31 | No estimate | | | | 2 | 0 | 0 |
| IL5 | 1.440 | 0.748 | 2.77 | 0.28 | 18 | 12 | 6 |

*Due to skewed nature of cytokines, HRs are reported on Log2 (log base 2) scale.

**Supplementary Table 5.** Association between body mass index and cytokines (parent cohort)

| | N | Mode | Median | Mean | Minimum | Maximum | Std Dev | Skewness | p-value* |
|---|---|---|---|---|---|---|---|---|---|
| **IL17a** | | | | | | | | | |
| BMI <25 kg/M$^2$ | 100 | 2.7300000 | 3.0400000 | 3.7922000 | 0.4400000 | 25.2500000 | 3.2608494 | 3.6157289 | |
| BMI 25-29.9 kg/M$^2$ | 138 | 1.6700000 | 3.1600000 | 3.4843478 | 0.2500000 | 12.7100000 | 2.0009884 | 0.9943973 | |
| BMI ≤30 kg/M$^2$ | 182 | 0.6300000 | 3.7250000 | 4.6024176 | 0.1200000 | 43.1100000 | 4.6578162 | 4.3052654 | 0.18 |
| **IL6** | | | | | | | | | |
| BMI <25 kg/M$^2$ | 113 | 0.280000 | 0.650000 | 0.790708 | 0.0400000 | 5.6900000 | 0.684393 | 4.118610 | |
| BMI 25-29.9 kg/M$^2$ | 152 | 0.570000 | 0.845000 | 1.275592 | 0.1700000 | 21.130000 | 2.024083 | 7.208629 | |
| BMI ≥ 30 kg/M$^2$ | 199 | 1.300000 | 1.240000 | 16.14422 | 0.1500000 | 2761.24 | 195.7234 | 14.07564 | <0.01 |
| **MDC** | | | | | | | | | |
| BMI <25 kg/M$^2$ | 130 | . | 961.5350 | 1015.56 | 392.86000 | 2493.25 | 373.2028 | 0.814683 | |
| BMI 25-29.9 kg/M$^2$ | 163 | . | 976.1100 | 1052.49 | 444.64000 | 2636.81 | 393.4601 | 1.161629 | |
| BMI ≥30 kg/M$^2$ | 205 | 1058.58 | 1061.17 | 1145.26 | 427.70000 | 4063.81 | 484.7726 | 2.273455 | 0.03 |
| **VEGFa** | | | | | | | | | |
| BMI <25 kg/M$^2$ | 125 | . | 69.35000 | 98.71408 | 0.6600000 | 422.34000 | 88.83385 | 1.674016 | |
| BMI 25-29.9 kg/M$^2$ | 157 | . | 81.78000 | 108.5057 | 2.0200000 | 460.49000 | 94.78010 | 1.452414 | |
| BMI > 20 kg/M$^2$ | 198 | 20.79000 | 77.33000 | 116.7970 | 0.8000000 | 1196.67 | 140.7761 | 4.380541 | 0.64 |

*based on Kruskal Wallis Test
