# Supplementary material for: Inflammatory cytokines and distant recurrence in HER2-negative early breast cancer
Source: NPJ Breast Cancer. 2022 Feb 8;8:16. doi: 10.1038/s41523-021-00376-9 (PMC8825796; doi:10.1038/s41523-021-00376-9)
Supplement: Supplementary file 2 — Reporting Summary [file 41523_2021_376_MOESM2_ESM.pdf]

nature portfolio

Corresponding author(s): Joseph Sparano

Last updated by author(s): Aug 23, 2021

Reporting Summary

Nature Portfolio wishes to improve the reproducibility of the work that we publish. This form provides structure for consistency and transparency in reporting. For further information on Nature Portfolio policies, see our Editorial Policies and the Editorial Policy Checklist.

Statistics

For all statistical analyses, confirm that the following items are present in the figure legend, table legend, main text, or Methods section.

n/a	Confirmed	
☐	☒	The exact sample size (*n*) for each experimental group/condition, given as a discrete number and unit of measurement
☐	☒	A statement on whether measurements were taken from distinct samples or whether the same sample was measured repeatedly
☐	☒	The statistical test(s) used AND whether they are one- or two-sided<br>*Only common tests should be described solely by name; describe more complex techniques in the Methods section.*
☐	☒	A description of all covariates tested
☐	☒	A description of any assumptions or corrections, such as tests of normality and adjustment for multiple comparisons
☐	☒	A full description of the statistical parameters including central tendency (e.g. means) or other basic estimates (e.g. regression coefficient) AND variation (e.g. standard deviation) or associated estimates of uncertainty (e.g. confidence intervals)
☒	☐	For null hypothesis testing, the test statistic (e.g. *F*, *t*, *r*) with confidence intervals, effect sizes, degrees of freedom and *P* value noted<br>*Give P values as exact values whenever suitable.*
☒	☐	For Bayesian analysis, information on the choice of priors and Markov chain Monte Carlo settings
☒	☐	For hierarchical and complex designs, identification of the appropriate level for tests and full reporting of outcomes
☒	☐	Estimates of effect sizes (e.g. Cohen's *d*, Pearson's *r*), indicating how they were calculated

Our web collection on statistics for biologists contains articles on many of the points above.

Software and code

Policy information about availability of computer code

Data collection	The analysis did not involve using custom code or mathematical algorithm
Data analysis	*Provide a description of all commercial, open source and custom code used to analyse the data in this study, specifying the version used OR state that no software was used.*

For manuscripts utilizing custom algorithms or software that are central to the research but not yet described in published literature, software must be made available to editors and reviewers. We strongly encourage code deposition in a community repository (e.g. GitHub). See the Nature Portfolio guidelines for submitting code & software for further information.

Data

Policy information about availability of data

All manuscripts must include a data availability statement. This statement should provide the following information, where applicable:

- Accession codes, unique identifiers, or web links for publicly available datasets
- A description of any restrictions on data availability
- For clinical datasets or third party data, please ensure that the statement adheres to our policy

The datasets generated during and/or analyzed during the current study are available in the NCTN/NCORP Data Archive (https://nctn-data-archive.nci.nih.gov)

Field-specific reporting

Please select the one below that is the best fit for your research. If you are not sure, read the appropriate sections before making your selection.

☒ Life sciences ☐ Behavioural & social sciences ☐ Ecological, evolutionary & environmental sciences

For a reference copy of the document with all sections, see nature.com/documents/nr-reporting-summary-flat.pdf

Life sciences study design

All studies must disclose on these points even when the disclosure is negative.

Sample size	266 case:control pairs with/without distant recurrence of breast cancer
Data exclusions	As described in Consort diagram (Figure 1)
Replication	This was a prospective:retrospectice training study done and reported in accordance with REMARK guidelines; no validation study in an independent cohort was performed.
Randomization	Patients enrolled in the parent trial all received adjuvant chemotherapy, and were randomized to receive adjuvant bevacizumab or not (1:2:2 - placebo vs. concurrent bevacizumab x 4 cycles plus chemotherapy vs. concurrent and sequential bevacizumab x 14 doses).
Blinding	Patients in the parent trial were blinded to bevacizumab/placebo during chemotherapy; unblinding occurred after completion of chemotherapy, and those randomized to bevacizumab x 14 cycles received open-label bevacizumab

Reporting for specific materials, systems and methods

We require information from authors about some types of materials, experimental systems and methods used in many studies. Here, indicate whether each material, system or method listed is relevant to your study. If you are not sure if a list item applies to your research, read the appropriate section before selecting a response.

Materials & experimental systems

n/a	Involved in the study	
☒	☐	Antibodies
☒	☐	Eukaryotic cell lines
☒	☐	Palaeontology and archaeology
☒	☐	Animals and other organisms
☐	☒	Human research participants
☐	☒	Clinical data
☒	☐	Dual use research of concern

Methods

n/a	Involved in the study	
☒	☐	ChIP-seq
☒	☐	Flow cytometry
☒	☐	MRI-based neuroimaging

Human research participants

Policy information about studies involving human research participants

Population characteristics	Covariates used in propensity score matches of cases (distant recurrence) and controls (no distant recurrence) included age, menopausal status, ER/PR expression, tumor size, axillary lymph node metastasis, and histologic grade.
Recruitment	Subjects with stage II-III breast cancer were recruited on NCI-sponsored treatment trial, provided a blood sample at time of enrollment, and consent to future unspecified use of data and blood sample
Ethics oversight	National Cancer Institute, local institutional IRBs

Note that full information on the approval of the study protocol must also be provided in the manuscript.

Clinical data

Policy information about clinical studies

All manuscripts should comply with the ICMJE guidelines for publication of clinical research and a completed CONSORT checklist must be included with all submissions.

Clinical trial registration	NCT00433511
Study protocol	Provided as supplementary material

Data collection	4994 patients accrued between November 2007 and February 2011
Outcomes	Primary outcome was time to distant recurrence
